# A Case of Immunotherapy-Induced Balanitis

**DOI:** 10.7759/cureus.94216

**Published:** 2025-10-09

**Authors:** Terrence Sun, Danielle Brazel, Aditya Sarvaria

**Affiliations:** 1 Hematology and Medical Oncology, Scripps Clinic, San Diego, USA

**Keywords:** balanitis, cancer immunotherapy, immunotherapy-related adverse events, lung cancer, pembrolizumab cutaneous side effect

## Abstract

Immunotherapy is indicated in a variety of different cancers with wide-ranging adverse events. Although dermatitis can be common, serious dermatologic complications (such as Stevens-Johnson syndrome) have also been reported. In this case report we present a case of balanitis caused by pembrolizumab, briefly review the current literature on more common dermatologic immunotherapy toxicities, and discuss the more common causes and treatments of balanitis. Although drug eruptions commonly respond to discontinuation of the offending agent, here we were able to treat with topical agents, such as steroid cream and mupirocin ointment. The ability to treat the balanitis without needing to discontinue the patient’s immunotherapy highlights the success in both controlling the cancer and the treatment-related toxicity.

## Introduction

Balanitis is a common urological condition, especially among uncircumcised men [1]. It involves inflammation of the glans penis and is diagnosed by physical examination. It commonly presents as penile soreness, dysuria, pruritus, and erythema of the glans penis [2]. It can be complicated by ulceration or extension of the inflammation. Although a physical examination may be sufficient to make the diagnosis for balanitis, a shave biopsy can be done to confirm the etiology and exclude malignancy [1].

In a typical case of new onset balanitis, it is important to consider poor personal hygiene as the etiology as that is the most common cause [1]. However, the differential diagnosis also includes drug eruption, infection, plasma cell balanitis, premalignant or malignant skin conditions, chemical irritants, allergic reaction, and trauma [1]. Immune checkpoint inhibitors are now being used more frequently in oncology, and are associated with a broad spectrum of immune-related adverse events (irAE's), with cutaneous manifestations being the most common [3]. Here, we describe, to our knowledge, the first documented case of pembrolizumab-associated balanitis, based on a literature search conducted using PubMed.

## Case presentation

**Figure 1:**
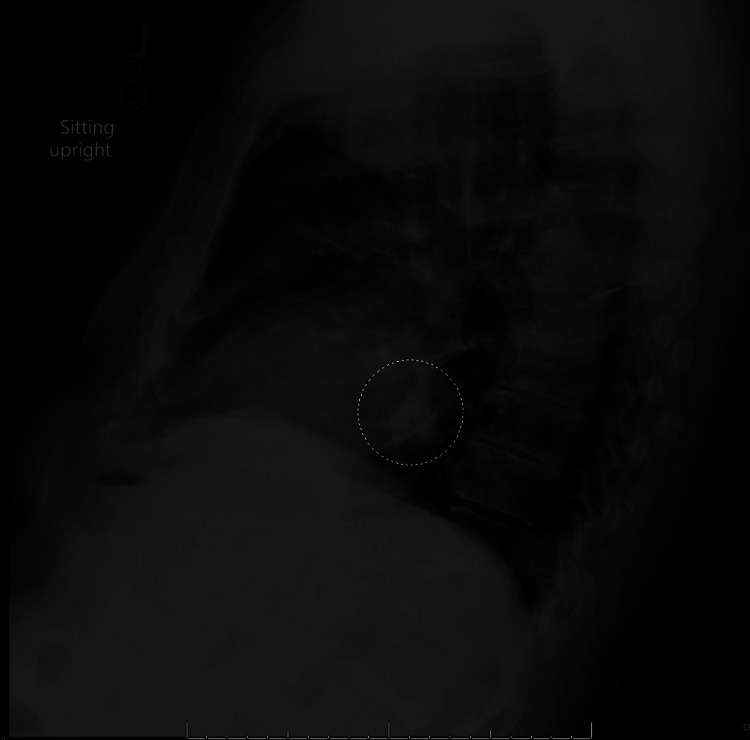
Chest X-ray with 2.5 cm infrahilar nodule

**Figure 2:**
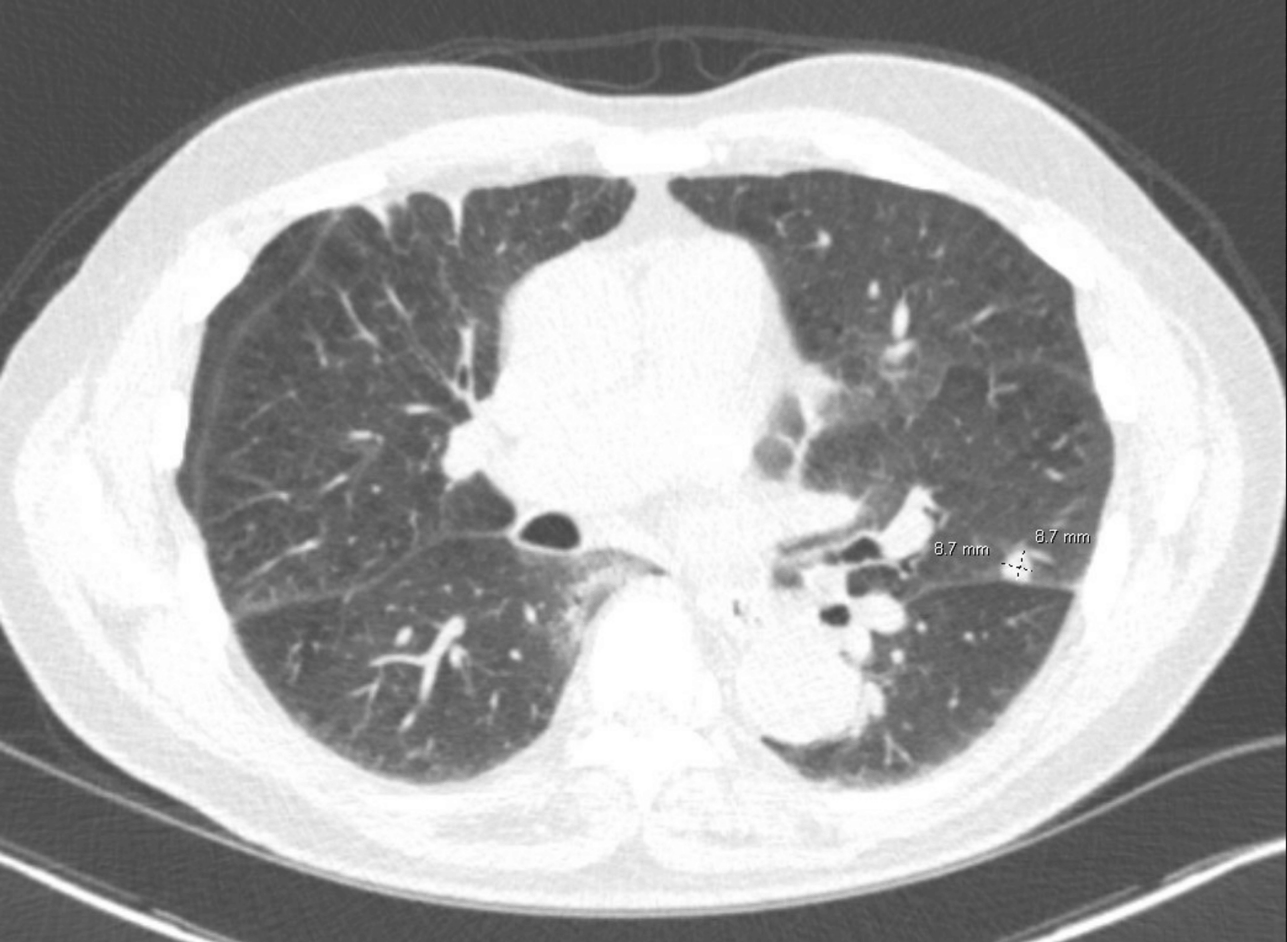
CT Chest with 9mm left upper lobe lung nodule

**Figure 3:**
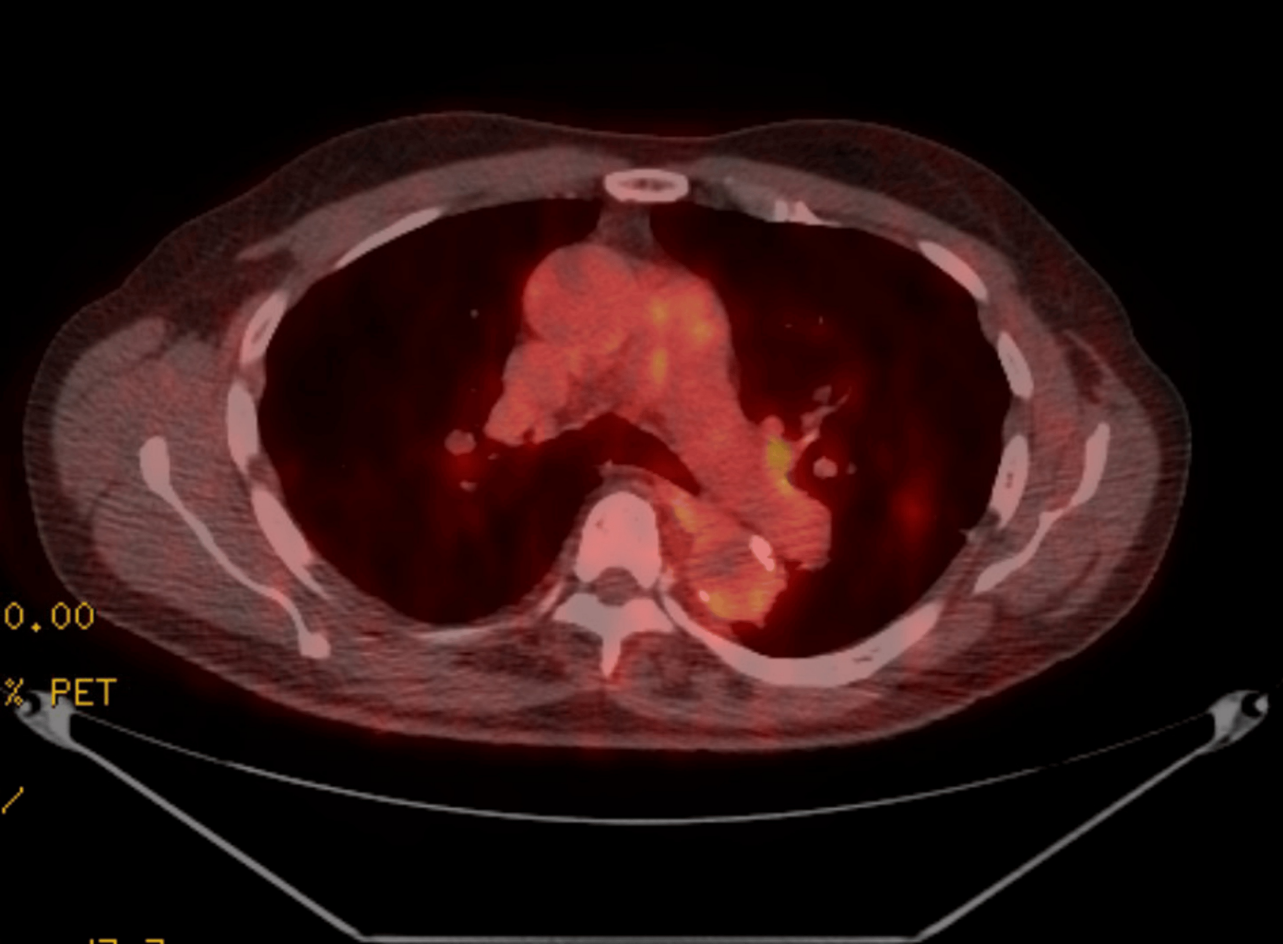
PET Scan with 1.1 cm nodule with mild to moderate uptake

An uncircumcised but otherwise hygienic male in his 70s was diagnosed with lung cancer in December 2019 when he presented to the emergency room with hypertension and a dry cough. Chest X-ray showed a 2.5-centimeter (cm) infrahilar nodule (Figure 1). January 2020 CT chest showed a 9-millimeter left upper lobe nodule (Figure 2). February 2020 PET CT scan showed a 1.1 cm nodule with mild-to-moderate uptake (Figure 3). Due to the COVID-19 pandemic, transthoracic needle biopsy was delayed until June 2020. Biopsy confirmed lung adenocarcinoma with micropapillary and acinar features. He underwent a lobectomy and lymph node dissection in September 2020. Pathology showed a T1N2 adenocarcinoma of the lung. 

He completed chemotherapy in December 2020. Unfortunately, after several years, his disease recurred with metastasis, so the patient was initiated on pembrolizumab monotherapy in March 2023. 

**Figure 4:**
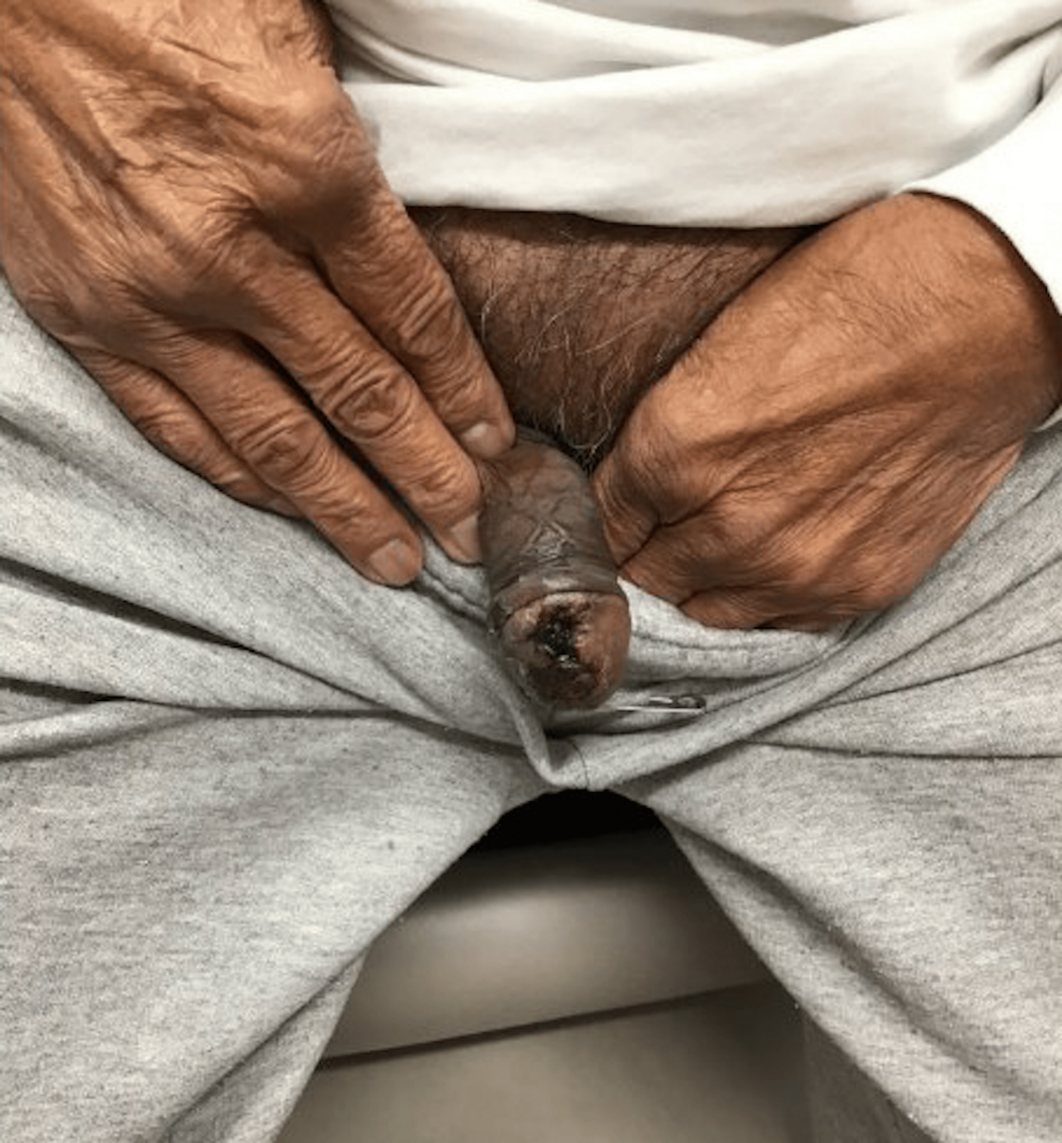
Patient’s initial diagnosis of balanitis in September 2023

In September 2023 the patient reported discomfort, difficulty with urination, and pruritus around his glans penis (Figure 4). He denied rash in any other location or any other new symptoms. In the emergency department, a physical exam revealed an eschar at the glans penis with some necrosis and pus. This would be considered a grade 3 dermatitis based on the Common Terminology Criteria for Adverse Events (CTCAE). Urinalysis was unremarkable. 

He underwent a shave biopsy of the lesion on the glans penis on October 4, 2023, which revealed squamous erosion with extensive serum crust formation. Grocott methenamine silver (GMS) and herpes simplex virus (HSV) stains were negative and gram stain showed commensal organisms. There were changes in the area consistent with spongiosis and lichenoid changes with dyskeratotic keratinocytes. The dermis also contained several hemosiderin laden macrophages, along with scattered neutrophils, lymphocytes and rare eosinophils. These findings were most consistent with lichenoid dermatitis secondary to a fixed drug eruption. 

**Figure 5:**
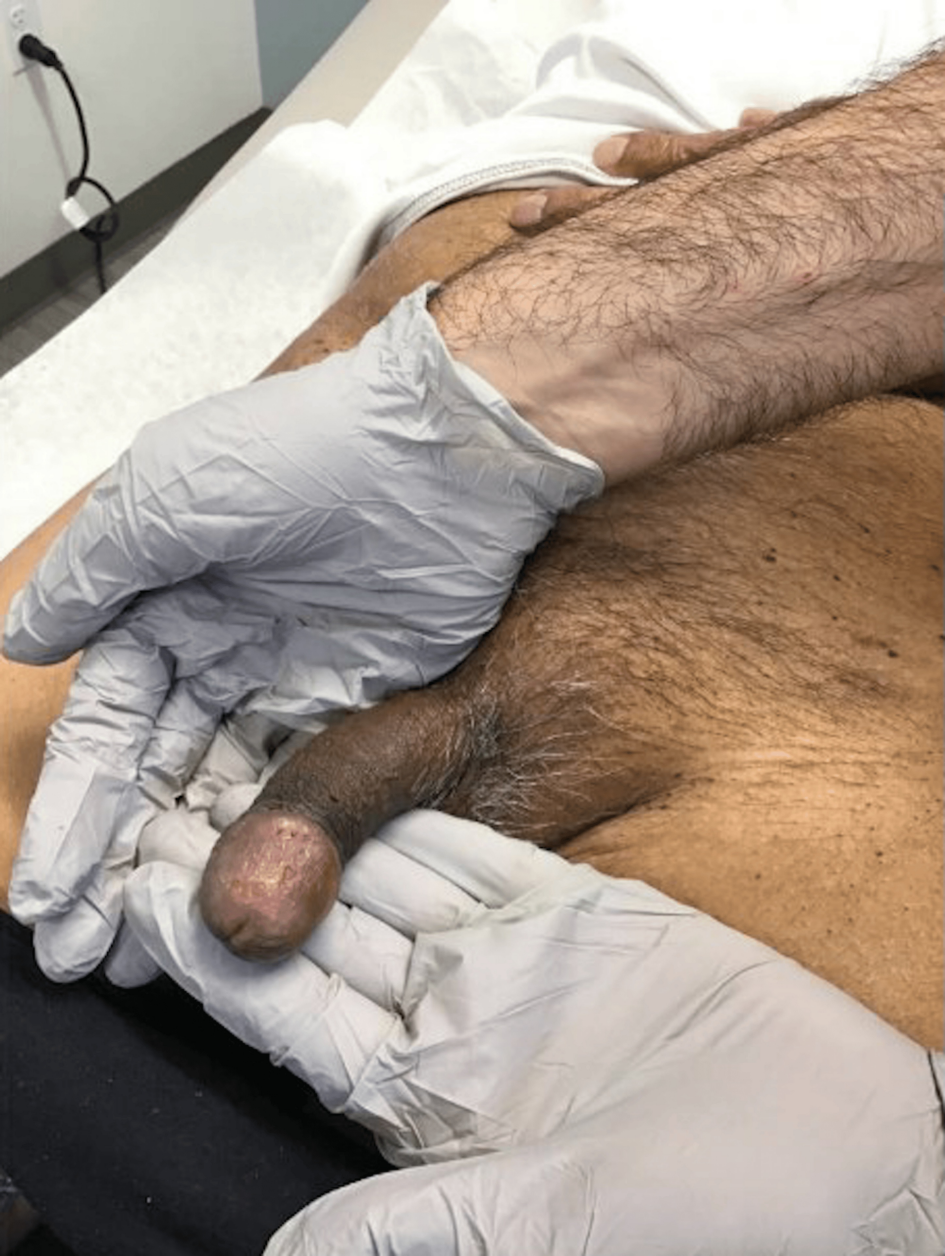
After treatment of patient’s balanitis in October 3, 2023

He was started on clobetasol 0.05% ointment and mupirocin 2% ointment four times per day, and since there was improvement in his symptoms at follow-up six days later (Figure 5), systemic therapy was deferred. With his improvement, he continued pembrolizumab monotherapy. Because of the concern for recurrence of balanitis from the immunotherapy, topical treatment was continued prophylactically for about two to three weeks after resolution of symptoms, but he has not had any recurrence since.

## Discussion

Our patient developed balanitis after about six months of treatment with pembrolizumab. Punch biopsy showed lichenoid dermatitis most consistent with a fixed drug eruption. He was treated with topical steroid and antibacterial ointments while continuing pembrolizumab with resolution of his symptoms.

Though this did not apply to this hygienic patient who had a negative infectious workup, balanitis is most frequently associated with infection secondary to poor personal hygiene. Common infectious etiologies include Candida, beta-hemolytic streptococci, Neisseria gonorrhoeae, Chlamydia, anaerobes, human papillomavirus, herpes simplex virus, varicella-zoster virus, Gardnerella vaginalis, monkeypox, Treponema pallidum, Trichomonas, and Borrelia [1]. Drug eruption is another frequent cause of balanitis [1]. Common medications associated with balanitis include tetracyclines, sulfonamides, 5-fluorouracil, and Bacillus Calmette-Guérin (BCG).

Review of the current literature regarding adverse effects of immunotherapy is extensive and often involves multiple organ systems, including skin, gastrointestinal, and the genitourinary tracts. One retrospective dataset study reported a case of grade 1 balanitis associated with nivolumab, another anti-programmed death ligand 1 (PD-1) agent [4]. Hoffman 2016 also mentions a case of lichen planus mucosae in addition to an erosion of the glans penis 49 weeks after initiation of pembrolizumab [3]. However, there is no documented correlation between pembrolizumab and balanitis.

Other dermatologic toxicities, such as toxic epidermal necrolysis [5] and Stevens-Johnson syndrome [6], are rare but have been documented in association with pembrolizumab. More commonly, anti-PD-1 agents are associated with many different dermatologic toxicities. In a study by Hofmann 2016, 496 patients with melanoma were treated with nivolumab or pembrolizumab. 8.7% of patients were reported to have dermatologic side effects [3]. These included pruritus, rash, and eczema (3.8%), vitiligo in 2.6%, alopecia in 1.4%, and lichenoid and cytotoxic skin reactions in 0.8%. Psoriasis vulgaris and lichen planus mucosae were reported in two patients each, and Sweet syndrome, lichen planus, and lichen sclerosus atrophicus were reported in one patient each [3]. However, there were no cases of balanitis that were mentioned in this article related to pembrolizumab. 

Balanitis is also a documented side effect of BCG treatment of transitional cell carcinoma of the urinary bladder [7-9]. The most common side effects of BCG include immediate hypersensitivity reactions and disseminated infections. Cutaneous side effects are rare but have been reported [7]. In one review, 14/15 (93%) of patients had complete resolution of their balanitis [7]. These adverse effects are attributed to the use of a live vaccine.

Typical treatment for balanitis associated with infection involves proper hygiene with frequent washing and drying, and empiric topical antifungal treatment [1]. Commonly prescribed topical antifungals include clotrimazole, miconazole, and nystatin. Drug-induced balanitis can be treated with topical moderate-to-high dose corticosteroids such as clobetasol 0.05%, betamethasone, or triamcinolone. This treatment was continued for about two to three weeks after resolution of balanitis; however, this is unique to this patient's case as there are no guidelines on the duration of prophylaxis in this specific situation. Oral steroids may be considered for more severe cases, and premalignant balanitis can be treated with topical 5-fluorouracil or topical imiquimod creams.

## Conclusions

To the best of the authors’ knowledge, this is the first documented case of balanitis secondary to pembrolizumab. In this patient who developed immunotherapy-associated balanitis, clobetasol and mupirocin showed significant improvement. This case highlights that pembrolizumab-induced balanitis, though previously unreported, can be effectively managed and allows for continuation of immunotherapy without treatment interruption. 
